# Life course approaches of women living with HIV in Matabeleland South Rural, Zimbabwe

**DOI:** 10.1177/17455057261455246

**Published:** 2026-07-29

**Authors:** Limkile Mpofu, Azwihangwisi H. Mavhandu-Mudzusi

**Affiliations:** 1 Department of Psychology, College of Human Sciences, 20381548 University of South Africa (UNISA), Pretoria, South Africa; 2 Department of Social Sciences, College of Human Sciences, 381547 University of Africa, Pretoria, South Africa

**Keywords:** Africa, diagnosis, HIV, life course approach, stigma and discrimination, and women

## Abstract

**Background:**

Women in Africa experience a disproportionate burden of HIV, with their life trajectories shaped by its health and social impacts. The variation is based on present psychosocial barriers and the stigma and discrimination they experience. These differences determine their life course approaches, shaping their health outcomes, mental health service engagement, and overall well-being.

**Objective:**

This qualitative study aimed to explore and describe the life space and life-span issues for women living with HIV to establish the policies that may support and explain women’s health-related quality of life.

**Design:**

An interpretative phenomenological design was followed in order to gain an in-depth understanding of the life courses of women living with HIV.

**Methods:**

Data were collected between December 2023 and May 2024. Semi-structured interviews were conducted in rural Matabeleland South Province with twenty (20) purposively sampled women living with HIV (aged 20-65) to explore their experiences and how HIV determines their life approaches. The Interpretive Phenomenological Analysis framework was employed for data analysis.

**Results:**

The findings indicated that women experience (i) Loss of control and planning, (ii) Deterioration in the quality of relationships, and (iii) limited access to education, training, and work. The younger women are the ones who are severely affected, as their daily activities and social roles tend to be disrupted while living in a state of social and economic insecurity. The only support received is from the peers who are also living with HIV in the form of a support group.

**Conclusion:**

Findings indicate the need for continuous psychosocial and financial support of young women living with HIV. Moreover, there should be a renewed emphasis on adherence to policies that promote non-discrimination.

## Introduction

Women in sub-Saharan Africa are disproportionately impacted by HIV, facing unique health, social, and economic challenges across the life course.^1^ Bury’s concept of biographical disruption states that ‘illness would interrupt the expectations and plans that individuals hold for the future.’ He posits that this would then require ‘a fundamental rethinking of the person’s biography and self-concept’^2^(p. 169). This fundamental rethinking is crucial for women who have proven that they are essential in HIV prevention efforts, because they are better caregivers than men. Besides, the high prevalence rate of HIV in the rural province of Matabeleland South (21,6% versus the 12.2% in Harare).^3^ Rural women (60%) produce agricultural commodities as they work 16 to 18 hours a day.^4^ Most of their time is spent on agricultural activities, of which 25 % is unpaid care work.^4^

Despite all the effort rural women put into agriculture, a report by Madrigal-Borloz^5^ on protection against violence says there are no efforts to undertake gender sensitization training for women, no provision of educational programs on women’s individual outcomes, and failure to address women’s property rights. FAO Zimbabwe^4^ also claims that about 68% of Zimbabwean women live in rural areas. At the same time, their life paths are determined by the expectations of their husbands, children, aging parents, and societal gender role norms.^6^ These women’s caregiver roles affect their psychological well-being because, while they desire to be socially independent, the actions of significant others may impinge on these women’s life approaches and choices.^7^ The Federation of Women Lawyers has also observed that the low socioeconomic female who is uneducated would be ignorant of the constitution that protects their land and property rights, resulting in the women not being able to claim the land and assets that they have rights over. Boyd and Bee^8^ say that women often receive emotional support from intimate partnerships, which enables them to cope with aging. Therefore, a look at the life space and life-span issues for women living with HIV is crucial in the study of women’s lives.

To understand the life course approaches of women living with HIV, the authors use Erikson’s stage of ego integrity versus despair theory because “ego integrity and despair are important indicators of life-span development”,^9^ p. 400. Existing studies in the Eriksonian tradition found that ego integrity would be achieved if an individual “comes to terms with who she is and has been, how her life has been lived, the choices she has made, and the opportunities gained and lost”.^9^ Settersten Jr and colleagues,^6^ on the other hand, say that significant vulnerabilities and encounters with illness and death would come with old age as the family members co-survive for long periods because of the reductions in mortality and fertility over the years.

Thus, the sociodemographic of women’s age would explain the differences in life outcomes of women living with HIV/AIDS. For example, women may also develop social care needs linked to mobility or physical health issues, such as the impact of dementia or related conditions. This would make the women diagnosed in later life struggle to adapt to and accept an unexpected HIV diagnosis.^10^ The HIV diagnosis presents social isolation and creates barriers to care and support.^11^ In summary, HIV would present social and psychological complications for women living with HIV as they face broader barriers linked to socio-economic and other factors. For these rural women, stigmatization created barriers to access testing, treatment, care, and support, as the socio-economic factors, including poverty and unstable housing, would impact adherence and treatment outcomes. However, lifelong health habits contribute to successful aging, but individuals’ responses to the health crises of old age also matter.^8^

Life course approaches of women living with HIV mean representing these rural women by age and life stages. Socially, it represents family and their actions which may impact women’s life trajectories and choices, cumulatively shaping women’s life courses and creating both inequalities and shared experiences. Bandura,^12^ p. 166) claims that “humans can take actions and make decisions, individually and collectively, that affect their life pathways and outcomes.” Thus, living or being affected by HIV may mean being affected by the social, economic, cultural, and psychological consequences of HIV.

In rural settings like those in Zimbabwe, universal health coverage (UHC) is lacking, resulting in health problems.^13^ To achieve Sustainable Goal 3 (ensure healthy lives and promote well-being for all ages), Kuper and colleagues think that Zimbabwe would need a global commitment to move towards UHC.^13^ Researchers have also highlighted that women’s health problems cannot be successfully addressed by medical care alone, meaning that living in poverty-stricken rural settings may present health risks for women, as a lack of nutritious foods may end up weakening their immune system.^14^ The life course and HIV emphasize the health risks of HIV depending on prior (and accumulated) biological, psychological, and social exposures. Primary health and disease conditions in adulthood and later life often have early developmental origins, even from the prenatal period.^15^

Examining the life course approach of these women living with HIV requires tracking the dynamics related to the growth, maintenance, and decline of physical and mental capacities of these women, as well as the onset and progression of chronic diseases and their preclinical intermediate phases.

Therefore, this research also accounts for the psychological consequences of being HIV positive, such as the fear and anxiety triggered by it, as well as the uncertainty of living with HIV for oneself or loved ones, now and in the future. Researchers, Derdaele and colleagues,^16^ posit that psychological issues among women would manifest if they failed to find meaning in their past life, as they would experience a sense of despair. Despair would kick in as they experience regret, bitterness, and disappointment over a life misspent. On the other hand, ego integrity would relate positively to mental health and to a lower risk for depressive symptoms, while despair would show an opposite pattern of associations.^16^ This despair would also make women develop anxiety as they get stigmatized in the society in which they grew up. Besides the stigma, the women would also face a loss of livelihood, as land inheritance is often given to a male child.^17^ In such a case, the bride would live with the husband’s family and forever owe them her primary allegiance.

### Goal of the study

In this article, the researchers present findings from a study exploring the life approaches of women living with HIV in rural Zimbabwe. The primary focus is on the life courses of these women, as this was identified in the experiences and narratives they shared in this research. The research reported on in this paper was driven by the following questions: What are the life-space and life-span issues for women living with HIV? What policy supports and explains the health-related quality of life for women living with HIV?

## Method

### Research design

The study utilized thematic analysis of women’s life stories. Life story interviews create space for a more balanced, wide-ranging, and participant-led interview than semi-structured or structured approaches.^18^ This was an interview about the life experience stories of rural women living with HIV. It included their past as they remembered it and their future as they imagined it. Such a story experience was selective as it did not include everything that had ever happened to them but focused on a few key scenes, characters, and ideas in their lives (see interview guide). In this research, we used the Standards for Reporting Qualitative Research checklist to organize our reporting (see Supplementary File 1).^19^

### Participants and setting

Recruitment for the life story experiences interviews was through the healthcare workers who purposively sampled twenty^20^ rural women living with HIV from Matabeleland South Province of Zimbabwe (See Table 1 for Characteristics of the study participants). The participants were selected if they could articulate their experiences with the phenomenon of interest.

**Table 1.** Characteristics of the study participants (N = 20).

Characteristics	N	%
Age, years		
20 to 27	2	(10)
28 to 35	2	(10)
36 to 43	6	(30)
44 to 51	7	(35)
52 to 59	3	(15)
Marital status		
Single/divorced/widowed	6	(30)
Married	14	(70)
Education level		
Primary or below	5	(25)
Secondary	8	(40)
Tertiary	7	(35)
Employment status		
Unemployed or retired	14	(70)
Employed	6	(30)
Income reliance		
Agricultural proceeds income	6	(30)
Employment proceeds	6	(30)
Support from relatives/Chn	8	(40)
Time since HIV positive and ART initiation		
+6 years	6	(30)
4–6 years	11	(55)
0–3 years	3	(15)

Source: Authors’ Own construction.

The following inclusion criteria were used to select eligible participants:

1) Be an adult woman (20-65 years) living with HIV.
2) Having tested HIV-positive for at least 1 year from the date of the data collection.
3) Being a permanent resident in the three selected villages of Matabeleland South Province for at least a year after testing HIV-positive.
4) Giving a good account of yourself in relationships, family, employment, and life in this village.

Such life story experiences from the women would ensure a range of experiences are reflected in the data.

The exclusion criteria consisted of all women living with HIV who were on treatment for any psychiatric condition. Qualitative IPA studies provided a deep exploration of the women’s personal experiences in this research. Hence, the principle of data saturation determined the sample size.

### Data collection

Data was collected over 6 months, from December 2023 to May 2024. These life experience story interviews were conducted in the participants’ homes and were guided and semi-structured with no right or wrong answer to the questions. No participant refused or dropped out. The women would tell the interviewers about their experiences with living with HIV, and how it has shaped them so far. These interviews took between 50 minutes and 120 minutes. However, the participants were assured that the interviews were not therapy sessions but only for research. The main goal was to hear their life stories and understand how people lived their lives and how these rural women understand their identities within their society. Sample questions are “How would you describe yourself? “How has HIV shaped or influenced your life?” “Do you feel you are in control of your life?” (See attached interview guide). The data was triangulated with the member checks and a subsample of the interviewees, and data from the audiotapes and the field notes were combined.

### Procedure and ethics

The study methods were approved by the human research ethics committees of the University of South Africa (UNISA) (HSHDC/847/2018) and the Medical Research Council of Zimbabwe (MRCZ) (MRCZ/A/2398). All data sources were well protected. The participants were given pseudonyms (codes) to maintain their privacy during data collection. The study respected participants’ autonomy, and so the women were given information about the study before they could participate. The researcher asked participants for permission before interviews were audiotaped. Only the researcher and the participant were present during the interviews. All participants signed informed consent. This was a voluntary participation, and no incentives were provided to the participants. All data were transcribed, anonymized, and stored in accordance with ethical guidelines. Transcripts were also checked for additional potentially identifying details to anonymize data.

### Data analysis

As in Mpofu and Ganga-Limando,^7^ this study employed the IPA method’s step-by-step transcript analysis, which involves organizing, coding, integrating, and interpreting data. The research followed the three main steps of analysis identified by,^20^ where the authors focused more on the subjective point of view of the participants than on factual data. Each participant’s transcript was read several times so the authors could familiarize themselves with the data and obtain a holistic overview of their thoughts and feelings through their stories. At each step, the authors recorded their observations and comments in this interpretative phenomenological analysis,^21^ to construct themes. To ensure the reliability of interpretations, a collaborator created a summary list of themes from the margin notes, developing the emergent themes using the transcripts. Differences were resolved through discussion to achieve a consensus view, allowing for both data collection and analysis to be fulfilled simultaneously until the interviews produced no new information, and theoretical saturation was reached.^22^

### Rigor and trustworthiness

This study used a piloted interview guide that was tested with four participants and revised based on their feedback (See Interview guide). The interviews were conducted by the first author, who has extensive experience in qualitative interviews with vulnerable populations.

The authors, both PhD female graduates, ensured rigor by detailing the sampling, data collection, and analysis processes. The two authors participated in the data analysis process, engaging in discussions to resolve coding differences and identify emerging themes. However, coding was done independently using a selection of transcripts.

In this study, the researchers bracketed their preconceptions, just as phenomenological studies do. Such bracketing would prevent preconceptions from influencing data collection and interpretation, thereby ensuring trustworthiness. When the study avoids subjectivity, the aim would be to capture the authentic meaning of the research respondents’ accounts.^23^

## Findings

Thematic analysis identified three themes, as in Table 1 below:

Each of these themes is discussed below with excerpts from the women participants.

### Theme 1: Loss of control and planning

HIV brought about the loss of control and rendered rural women powerless in the planning of their lives as they were treated as reproducers, caregivers, sexual outlets, and agents of family prosperity by their families. Such loss of control could lead to disengagement from important life goals, as seen from the following narrations by these women:

*I have lost control of my life. I do not think about getting old, as I might not get to see my grandchildren even with these antiretroviral drugs*. (Respondent # 02, 42 years old).

Society did not care about women’s virtues of submission, which were meek, self-effaced, or full of sacrifice, so that even in the event of the death of their husband, they would be sent away to their parents’ home, even though the women would have remained devoted to their husbands. A 44-year-old married woman living with HIV, who was sent back to her village by her in-laws after the death of her husband, narrated:

*My future is bleak. I came home after my husband's death, but did not even have the energy to think about tomorrow. I have lost it all. I just let them plan my life*. (Respondent # 19, 44 years old).

These women would mourn their husbands dearly, but they would be cast out and even lose material possessions after the husband’s death. They are forcefully sent back to their own parents’ homes. This is what a 39-year-old woman said about her experience after the death of her husband.

*Three weeks after my husband died of HIV and AIDS-related illness, there were family squabbles between my people and my matrimonial family about possessions. My family came and took me back home and said that they had to take me away from my husband's family for protection.* (Respondent # 20, 39 years).

Again, women living with HIV were socially stigmatised as they faced gender inequalities and were put behind the scenes and denied decision-making roles at social gatherings. A 41-year-old woman who has belonged to a women’s club for years talked of her experience with this club after disclosing her HIV-positive status.

*The changes that happened when I told club members of my HIV-positive status were unbelievable. They excluded me from all decision-making processes.* (Respondent # 15, 41 years).

The traditional view of women as family kin keepers and care providers makes interdependence meaningful in studying women’s lives. Thus, some women formed support groups that increased their participation in the life course. Support groups positively affected women, integrating them socially and helping them exercise a sense of control and improve their health, as they were also able to plant gardens. A 40-year-old woman narrates as follows:

*My only friends now are people we attend the support groups with, as people living with HIV. Here, I feel wanted, as we have established gardens to plant various vegetables for sale to the town. We also have established some markets to sell our produce* (Respondent # 06, 40 years).

Moreover, the differences by socioeconomic status are likely to be substantial if family resources significantly influence women’s lives and limit their control and choices.

#### The following narrative from a 50-year-old woman bears

*It pays to be born into a higher socio-economic stratum. I have been privileged to be born into a family with better economic standing*. *Even with HIV, I am advantaged because I go around educating the newly diagnosed on how to live with HIV.* (Respondent # 04, 50 years).

### Theme 2: Deterioration in the quality of relationships

Although rural women constitute a significant portion of the global population, society sometimes shuns them, leaving them wanting to belong and form social relationships. These women would engage in various agricultural practices and unpaid care work, serving as the primary workforce in rural communities, which are essential to both community and national economies; however, loneliness would affect their physical health, leading to feelings of isolation and despair. Women are crucial in managing agricultural activities and household responsibilities, contributing to food security and overall well-being. The following excerpt is from a 57-year-old woman lamenting her HIV positive status and loneliness:

*HIV impaired my mobility. I cannot visit my friends, and they do not come either. I am alone (Respondent #04, 57 years old).*

The deterioration of quality relationships was accompanied by reduced economic resources for rural women living with HIV. The absence of a male child meant they had no land rights, under their culture. The women were voiceless. The following statement is from a 47-year-old woman farmer.

*HIV silenced us. You cannot express your view. No matter the length of time you would have stayed with your husband, his relatives would strip you of everything you sweated for with your husband.* (Respondent #02, 47 years old).

In some instances, the women felt their lack of knowledge about land matters led to a deterioration in relations, as they felt oppressed by the system of their society.

*I feel we are shortchanged as widows because if you report to the chief or his assistant on land matters, he will not take my side but will side with what the community wants. They are too biased. They collude against us, and the government is not truthful to their word as they do not help us, the widows (Respondent # 01, 61 years).*

In this study, unmarried women experienced a postponement in childbearing or marriage due to several factors related to resources and uncertainties, as these were potential sources of stigmatization.

*The society views us as vectors of HIV infection, and they would show disdain towards those who want to have children while living with HIV (Respondent # 20, 37 years).*

In the narration of their life histories, some women indicated that once they had tested positive for HIV, it meant they could not be allowed to bear children. Bearing children while living with HIV signified cruelty by the mother.

### Theme 3: Education, training, and work

This theme examines the socio-demographic factors underlying women’s educational levels by looking at the differences in life outcomes among these women. A 49-year-old married woman living with HIV, who has only girls, shared her experience on how it affected their life after the death of her husband.

*My husband passed away five years ago, and I only have girls who cannot fend for me because they cannot find decent work with no education* (Respondent # 11, 49 years).

Another typical account came from a 22-year-old woman who was studying at a tertiary institution when she tested HIV positive. After disclosing her HIV status, the siblings who were paying her fees refused to pay and arranged for her to marry an older man. Her brothers had speculated that she would not have a promising future while living with HIV.

*I was in my first year of university when I tested positive for HIV. After sharing the news with my legal guardian, who was paying for my studies, he told me to return home. He also forced me to marry an older man.* (Respondent #03, 22 years old).

Some women narrated that their employment opportunities shrank the moment they tested HIV positive, as it meant that work opportunities were curtailed by their HIV-positive status. This had lifelong consequences as time was ticking on, eventually exposing the women to additional economic demands of parenthood.

A 49-year-old woman narrated her experience of being demoted after disclosing her HIV-positive status to her boss.

*Being HIV-positive has left a lasting mark on my income*. *I told my boss about my HIV-positive status, hoping to get the necessary support from him. Instead, he reduced my working hours and demoted me, even though I was not sick, as I performed my usual duties flawlessly.* (Respondent #10, 49 years old).

## Discussion

The present study explored and examined Zimbabwean rural women’s past life events to find meaning and reconcile positive and negative experiences. The women’s narratives portray their lives as limited in possibilities, leading to despair. Women ascribe their HIV positive status to limits that are relative to the larger socio-cultural context. (Table 2) They engage in sense-making processes that relate to the cause of their infection, social stigma, and the role of family and community, while seeking to be heard.^24^ While society expects women to be capable, economically productive, qualified, and skilled, it also simultaneously ascribes them to patriarchal norms.^24^

**Table 2.** Summary of themes with quotes and key findings.

Theme	Representative Quotes	Key Findings
Theme 1: Loss of control and planning	*I have lost control of my life. I do not think about getting old, as I might not get to see my grandchildren even with these antiretroviral drugs. (Respondent # 02, 42 years old).*	The women lost control of their life. Despite being loyal and submissive to their husbands and matrimonial home, they were shunned, disregarded and had their lives being planned by other people and subjected to squabbles between the women’s people and those of her matrimonial home. Women living with HIV were subjected to undignified treatment and gender stereotypes, with no decision-making roles, to render them hopeless.
	*My future is bleak. I came home after my husband’s death, but didn’t even have the energy to think about tomorrow. I have lost it all. I just let them plan my life. (Respondent # 19, 44 years old).*	The only support received was from the peers who are also living with HIV in the form of a support group.
	*My only friends now are people we attend the support groups with, as people living with HIV. Here, I feel wanted as we have established gardens to plant various vegetables to sell to the town. We also have established some markets to sell our produce (Respondent # 06, 40 years old).*	In some cases, the socio-economic stratum determined their social standing in their society
	*It pays to be around and born in higher socio-economic strata. I have been privileged to be born into a family with better economic standing. Even with HIV, I am advantaged because I go around educating the newly diagnosed on how to live with HIV. (Respondent # 04, 50 years old).*	
Theme 2: Deterioration in the quality of relationships	*HIV impaired my mobility. I cannot go around visiting my friends, and they don’t come either. I am alone in this world (Respondent # 04, 57 years old).*	Since living with HIV was accompanied by reduced economic resources, this was aggravated by loss of land rights which was further made worse by having no male heir. Quality relations diminished even though the women would have been loyal to their husband throughout their lives.
	*HIV silenced us. You cannot express your view. No matter the length of time you would have stayed with your husband, his relatives would strip you of everything you sweated for with your husband. (Respondent #02, 47 years old).*	The women in this study were stripped of their possessions after losing their husbands. They felt the system oppression as they also lacked knowledge in land matters.
	*I feel we are shortchanged as widows because if you report to the chief or his assistant on land matters, he will not take my side but will side with what the community wants. They are too biased. They collude against us, and the government is not truthful to their word as they do not help us, the widows (Respondent # 1, 61 years old).*	The unmarried women living with HIV in this study, would feel lower self-esteem as they are disdained if they developed a desire to bear children while living with HIV. Such negative outcomes (lower self-esteem, self-efficacy, and recovery orientation) caused the women greater psychological stress resulting in the deterioration of relations as they self-isolated themselves and withdrew from society.
	*The society views us as vectors of HIV infection, and they would show disdain towards those who want to have children while living with HIV (Respondent # 20, 37 years old).*	
Theme 3: Limited access to education, training, and work	*My husband passed away five years ago, and I only have girls who cannot fend for me because they can’t find decent work with no education (Respondent # 11, 49 years old).*	In this society, the girl child is isolated and denied educational or training opportunities due the lack of support for a girl child. In the end, the girl child who in an African culture, is likely to fend for the mother has limited capacity to do so because of lack of decent job and training.
	*I was in my first year of university when I tested positive for HIV. After sharing the news with my legal guardian, who was paying for my studies, he just told me to come back home. He also forced me to marry an older man. (Respondent # 03, 22 years old).*	The girl child is also marred off forcefully to older man because they are living with HIV.
	*Being HIV-positive has left a lasting mark on my income. I told my boss about my HIV-positive status, hoping to get the necessary support from him. Instead, he reduced my working hours and demoted me, even though I was not sick, as I performed my usual duties perfectly. (Respondent # 10, 49 years old).*	Again, at workplaces, once the bosses learnt of the individual’s HIV status, they would be demoted, making things tight for women living with HIV.

Source: Authors’ own construction.

Therefore, the loss of control and planning meant reduced food security, an important barrier to antiretroviral treatment adherence, as it causes some socioeconomic differences in the effects of HIV for the rural women,^25^ because women living with HIV face intersectional stigma, domestic violence, and marginalized social identities. According to Mukerji and colleagues,^25^ these interact to amplify stigma and related violence. These differences among older people reflected advantages and disadvantages that had accumulated over the life course.^26^ Those women with family and work responsibilities viewed HIV as acute as they would shoulder the responsibilities of supporting young adult children and caring for older parents. The literature also stipulates that when women reach midlife, their time horizons shorten, leading them to channel all their efforts into building security for later years and pursuing goals and experiences that bring meaning, thereby avoiding despair and disappointment.^27^ Thus, older adult women will likely feel a significant loss of control and despair.^28^ Researchers^9^ have also labelled ego integrity and despair as crucial later-life features related to previous quandaries.

They say that women with lower socioeconomic status (SES) have fewer resources to redirect their lives because their control capacity and self-efficacy are only possible when one is healthy, wealthy, and has intact social relationships. Human beings yearn to interact with others and feel a sense of belonging in their social relationships.^29^ Loneliness arises when an individual does not experience the intimacy commonly associated with interactions.^30^ Loneliness impacts women’s physical health,^31^ creating feelings of isolation and despair.^32^ Older women living with HIV in this study focused more on the present than the future. Their worry was more about social support and feeling wanted by their relatives or significant others. Other researchers have also expressed the same feelings about women, claiming that they desire immediate joys that uplift their daily lives and long for relationships with close family members and friends.^33^ This may augment a positive attitude for the women and minimize negative affective experiences.

With the HIV epidemic, the authors observe disruptions or alterations to life approaches for women.^34^ In African culture, for example, children often form a strong bond. With no bond, the African system is disrupted.^35^ Again, such changes may affect the community’s population structure.

Living with HIV meant that young adult women had to scale back their educational aspirations; they were labelled and discriminated against because they had tested HIV positive.^36^ Thus, the delays in women’s educational transitions affect women’s life courses indirectly as they alter their employment chances, thereby delaying their transition to adulthood.^37^ This makes these women rely on their parents or caregivers. A transition to growth then requires young adults to be re-trained and upskilled to make the policy that supports the health-related quality of life for these women living with HIV, to improve their lives. Such policies would help moderate unemployment challenges, as they can be implemented at the workplace or family level. Family policies would mediate gender, race, and other inequalities to render care a duty for both females and males.^38^ Besides, it relieves women of caring for the sick alone.

The HIV epidemic reshaped transitions, adulthood, and trajectories in every domain of life and instigated turning points that redirected women’s lives.^10^ This is consistent with Rochon and colleagues, who advocate for a supportive environment that encourages women’s participation in leadership positions and that encourages women in medicine to take on academic leadership positions.^39^ Again, some barriers hinder some widows from exercising their land rights. If the Government conscientizes them about the progressive steps to secure their land rights, such policies would reshape their transitions and instigate a turning point for the widows.

Again, life has its own risks, and HIV heightens the trends that affect educational transitions, family formation, and general trust within families.^40^ While there may be insufficient savings throughout a woman’s life course, this can undermine the relevance of life-course analysis, interventions, and policies. The HIV epidemic has isolated people from one another and created divisions.^41^ However, it can be said that it has also fostered a sense of collective solidarity, community action, cooperation, and the inherent need for mutual support, such as with support groups.^42^ It exposes inequalities in life course processes and outcomes, differentially affecting groups based on age, gender, race and ethnicity, social class, and other social categories.

Overall, while HIV/AIDS has been unveiled, the socioeconomic, ethnic/racial, and gender inequities are magnified by existing income and health inequalities.^43^ To foster or hinder recovery and the redistribution of resources toward the most vulnerable, specific policies related to the economy, work, education, and health can be beneficial. From the above findings, we note that some women had lost their employment due to HIV, which means that these policies would protect those women who have lost their employment, income, or hope.

Although being stigmatized for living with HIV can turn into permanent scars, women’s experiences may reorient their resilience and approach their lives with renewed energy.^3^ Looking forward, a life course perspective also encourages researchers to understand the HIV-related stigma effects. This would enable them to examine these dynamics at both the individual and group levels.

The findings suggest the need to strengthen psychosocial support through rural peer groups and community health workers to reduce stigma and isolation. Tailored, life-course-sensitive programming should address evolving needs from adolescence to older adulthood, while mobile clinics and confidential counseling can help overcome geographic barriers. Finally, integrating HIV care with gender-sensitive economic empowerment initiatives is essential to tackle structural inequalities and improve women’s quality of life, just as in the studies by Holmes.^44^

## Limitations

This study has several limitations. As this study employed an Interpretative Phenomenological Analysis (IPA) design, it provided deep insights into women’s lived experiences, but its reliance on a small, purposively selected sample limited generalizability beyond the study setting.^45^ The idiographic focus privileged depth over breadth, leaving broader structural determinants such as poverty, gender inequality, and gaps in the health system less explored. Furthermore, cross-sectional data collection constrained the life course perspective, while social desirability bias may have led participants to underreport sensitive issues like stigma, discrimination, or treatment non-adherence.^46^ Additionally, the presented temporal constraints are a limitation, as life course perspectives require longitudinal engagement; however, this study captured experiences cross-sectionally. As such, evolving trajectories of stigma, resilience, and health-seeking behaviors across different life stages remain underexplored.

### Future recommendations

The findings highlight several areas for policy and practice improvement, such as establishing psychosocial support through rural peer-support groups and community health worker programs to reduce isolation and stigma. Life course-sensitive programming could also help tailor interventions to different stages of women’s lives, recognizing evolving needs from adolescence to older adulthood. Geographic barriers could also be alleviated by expanding mobile clinics and confidential counselling services. Gender-sensitive policies can also enable the Integration of HIV care with economic empowerment initiatives to address structural inequalities.

### Implications for future research and practice

The life course approach for women living with HIV reveals the epidemic as having heightened the inequalities among families, as these families have varied resources available to them.^47^ Those with greater resources can support and ensure their children receive proper education. Therefore, this study has implications for educational policy and for reducing inequalities among children, youth, and young adults. Again, being HIV positive may have a detrimental effect on the one living with HIV, as it decreases their desire to improve themselves educationally.^48^ On the other hand, the parents or caregivers might not see the need to pay for their children living with HIV.^49^ This may impact the job opportunities of those children, as several jobs require higher educational attainment.^50^ In this study, women living with HIV, particularly those depending on their parents or siblings for support with their studies, were excluded from decision-making pertaining to the way forward with their own advancement in education after they had tested HIV positive.

The life course of women is crucial, as it helps us understand the transition to caregiving and its implications for quality of life. The women’s prior caregiving and the resources available to them would shape the impact of caregiving and women’s well-being.^51^ Besides, the caregiving transition lays a foundation for a life-course framework, linking the roles, resources, and life experiences to women’s well-being.

Erikson’s stage of ego integrity versus despair theory and the life course paradigm have shown their significance in investigating women’s life course, which primarily revolves around caregiving and psychological well-being.

## Conclusion

Above all, the life course approaches of these women living with HIV have created a powerful lens to understand their experiences of living with HIV in rural areas of Zimbabwe. At the same time, it invites researchers to look beyond historical time and to consider women’s personal histories as they intersect with their social histories. The health risks of living with HIV relate to these women’s age. The HIV epidemic has disrupted these women’s daily activities and social roles, while also heightening the young adults’ social and economic insecurity.

## Supplemental material

Supplemental material - Life course approaches of women living with HIV in Matabeleland South Rural, Zimbabwe Supplemental material for Life course approaches of women living with HIV in Matabeleland South Rural, Zimbabwe by Limkile Mpofu, Azwihangwisi H. Mavhandu-Mudzusi in Women’s Health.

## Data Availability

The data is available on request from the first author.*
