# Supplementary material for: Life course approaches of women living with HIV in Matabeleland South Rural, Zimbabwe
Source: Womens Health (Lond). 2026 Jul 29;22:17455057261455246. doi: 10.1177/17455057261455246 (PMC13424773; doi:10.1177/17455057261455246)
Supplement: Supplemental material - Life course approaches of women living with HIV in Matabeleland South Rural, Zimbabwe [file sj-pdf-1-whe-10.1177_17455057261455246.pdf]

Life course approaches of women living with HIV in Matabeleland Rural Areas, Zimbabwe

What are the life-space and life-span issues for women living with HIV?

- Tell me about your life experiences as a woman living with HIV in this village?
  What does it mean to you as a woman living with HIV in this village?
  Probe: How has living with HIV shaped your life in this village?
- How would you describe yourself?
- How has HIV shaped or influenced your life?
- Do you feel you are in control of your life?
- What kind of help or support do you receive from any other person, such as families, friends, and neighbors? Probe: Can you describe the quality of relationships you have?
- Describe how accessible education, training, and work are now that you were diagnosed with HIV. Probe: Are there any changes in the treatment as compared to before you disclosed your HIV status?
- Are you working at the moment? Can you describe the type of income you were receiving before testing HIV positive? Probe: Were you employed, training, or doing any course before you tested HIV positive?
- What made things difficult for you after testing HIV positive?
- Describe what would have made things easier for you while living with HIV in this rural setting?

What policy supports and explains the health-related quality of life for women living with HIV?

- What do you think can help alleviate stigma and discrimination in your village?
- Are you aware of any policies that can help you psychosocially or financially while living with HIV?
- Describe what you need to do and where to go if you face any stigma and discrimination from your society?
